# Mean Corpuscular Volume Is Not a Reliable Predictor of Iron Deficiency in Patients With Chronic Kidney Disease: A Post-Hoc Analysis of the BRIGHTEN Trial

**DOI:** 10.7759/cureus.83159

**Published:** 2025-04-28

**Authors:** Sayaka Shimizu, Tatsuo Kagimura, Shoichi Maruyama, Ichiei Narita

**Affiliations:** 1 Department of Research, Patient Driven Academic League (PeDAL), Kyoto, JPN; 2 Section of Clinical Epidemiology, Department of Community Medicine, Graduate School of Medicine, Kyoto University, Kyoto, JPN; 3 Translational Research Center for Medical Innovation, Foundation for Biomedical Research and Innovation, Kobe, JPN; 4 Department of Nephrology, Nagoya University, Aichi, JPN; 5 Division of Clinical Nephrology and Rheumatology, Niigata University Graduate School of Medical and Dental Sciences, Niigata, JPN

**Keywords:** anemia, diagnosis, iron-binding proteins, iron deficiency, prognosis, renal insufficiency

## Abstract

Introduction

Timely diagnosis of iron deficiency is crucial for managing anemia in patients with non-dialysis-dependent chronic kidney disease (ND-CKD). However, iron status results are not always immediately available. We aimed to examine the performance of mean corpuscular volume (MCV) in diagnosing iron deficiency as a primary objective and of iron status in predicting the onset of iron deficiency as a secondary objective.

Methods

This retrospective cohort study included adult patients with ND-CKD from the BRIGHTEN (oBservational clinical Research In chronic kidney disease patients with renal anemia: renal proGnosis in patients with Hyporesponsive anemia To Erythropoiesis-stimulating agents, darbepoetiN alfa) trial (enrolled from June 2014 to September 2016) in Japan who were started on darbepoetin-alpha for renal anemia and patients who were not started on iron supplementation during weeks 0-24. Diagnostic performance analysis assessed the MCV at week 24 and changes in MCV from week 12 to 24 for diagnosing iron deficiency (ferritin < 100 ng/mL) at week 24, using receiver operating characteristic curves, area under the curve (AUC), sensitivity, and specificity. For the patients without iron deficiency at week 12, ferritin levels at week 12 were assessed by predictive performance analysis to predict new-onset iron deficiency at week 24.

Results

A total of 796 participants were included in the diagnostic performance analysis (mean age, 70.0 years; mean estimated glomerular filtration rate, 19.2 mL/min/1.73 m²), and 338 were included in the predictive performance analysis. Diagnostic performance analysis revealed an AUC for MCV of 0.55 (95% confidence interval (CI), 0.51-0.59); for MCV changes, it was 0.52 (95% CI, 0.48-0.57). Prognostic performance analysis revealed that ferritin at week 12 demonstrated an AUC of 0.77 (95% CI, 0.67-0.86), with a sensitivity of 83% and a specificity of 66% at a cutoff of 131.5 ng/mL.

Conclusion

Neither MCV nor the changes in MCV could reliably diagnose iron deficiency in patients with ND-CKD. Ferritin level < 130 ng/mL could predict new-onset iron deficiency within 12 weeks.

## Introduction

Chronic kidney disease (CKD) is defined as kidney damage or an estimated glomerular filtration rate (eGFR) of <60 mL/min/1.73 m² for ≥3 months [1]. Kidney damage includes proteinuria, urinary sediment abnormalities, pathologic abnormalities suggested by kidney biopsy, or imaging abnormalities. CKD is highly prevalent, with a global median prevalence of 9.5% [2].

Anemia frequently coexists with advanced CKD, and its primary etiology is reduced production of erythropoietin, a hormone that promotes erythropoiesis by inhibiting apoptosis of erythroid progenitors by the kidney [3]. Additionally, patients with advanced CKD often develop iron deficiency anemia, which can be classified into two types: absolute iron deficiency and functional iron deficiency [4]. Absolute iron deficiency is characterized by depleted total body iron stores. In patients with CKD, factors such as blood loss from frequent testing, gastrointestinal losses exacerbated by anticoagulants or antiplatelets, reduced iron absorption, and inadequate dietary intake contribute to negative iron balance. Functional iron deficiency is characterized by normal or elevated iron stores, but iron availability is insufficient to support effective erythropoiesis, primarily due to inflammation. Thus, the assessment and correction of iron deficiency, along with the use of erythropoiesis-stimulating agents (ESAs), are key aspects in the management of anemia in patients with CKD.

While bone marrow aspiration provides a definitive diagnosis of iron deficiency, serum ferritin level and transferrin saturation (TSAT) are commonly used as surrogate markers [5-7]. However, the results of these tests are not always available on the same day, or these markers are not routinely measured. Early diagnosis of iron deficiency may be more challenging for patients with non-dialysis-dependent CKD (ND-CKD) than for those on hemodialysis because of fewer visits. The mean corpuscular volume (MCV), which represents the average size of RBCs, is frequently measured in complete blood counts (CBCs) and aids in the differential diagnosis of anemia (microcytic, normocytic, or macrocytic). A systematic review including a broad spectrum of participants showed that an MCV ≤70 fL had a high diagnostic performance for iron deficiency [5].

In patients with CKD, the MCV tends to be larger owing to factors such as age [8], ESA-induced reticulocytosis [9], and reduced crystal osmotic pressure due to malnutrition [10]. Anemia in CKD is also typically normocytic, even in the presence of iron deficiency [11]. Given these findings, alternative MCV cutoffs may better identify iron deficiency in patients with CKD and anemia. However, previous studies have been descriptive, with small sample sizes, and limited to patients on dialysis [11,12].

Therefore, this study aimed to investigate the diagnostic performance of MCV in detecting iron deficiency in patients with ND-CKD and anemia. As a secondary objective, we explored the cutoff values of ferritin and TSAT for predicting new-onset iron deficiency and, thus, provide guidance for timely iron supplementation.

## Materials and methods

Study design and setting

This study used data from the oBservational clinical Research In chronic kidney disease patients with renal anemia: renal proGnosis in patients with Hyporesponsive anemia To Erythropoiesis-stimulating agents, darbepoetiN alfa (BRIGHTEN) trial, which was a multicenter prospective cohort study conducted to evaluate ESA resistance to darbepoetin alfa (DA) and its associated risk factors in treating anemia in ND-CKD in a clinical setting. The design of the BRIGHTEN trial has been previously described [13]. Briefly, patients with ND-CKD, defined as an eGFR < 60 mL/min/1.73 m², aged ≥ 20 years, and who presented with renal anemia (hemoglobin < 11 g/dL) were enrolled from June 2014 to September 2016. Patients were excluded if they were scheduled to initiate kidney replacement therapy within 24 weeks after registration; had a history of ESA treatment; had malignant tumors, hematologic disorders, or hemorrhagic diseases; or had known hypersensitivity to ESA or its components. DA was administered according to the package insert, and the patients were observed for 96 weeks after administration. Iron supplementation was administered without restrictions. The administration status of DA, iron, and other drugs; body weight; blood pressure; and laboratory tests were examined and recorded at weeks 2, 4, 6, 8, 10, 12, 16, 24, 36, 48, 60, 72, 84, and 96. Regarding laboratory data, hemoglobin and MCV were measured by CBC; ferritin by the chemiluminescent enzyme immunoassay; and serum iron (Fe) and total iron-binding capacity (TIBC) by the nitroso-PSAP method.

This study was conducted in accordance with the principles of the Declaration of Helsinki and the Ethical Guidelines on Clinical Studies of the Ministry of Health, Labor and Welfare of Japan. Written informed consent was obtained from all the participants. The protocol of the BRIGHTEN trial was approved by the main institutional review board (Nagoya University, no. 2014-0027) and each participating facility. The study was registered at ClinicalTrials.gov (NCT02136563) and UMIN-CTR (UMIN000013464). This post-hoc analysis of the study was approved by the institutional review board of Niigata University (no. 2023-0014).

Participants

Among patients with ND-CKD and renal anemia who were eligible for the BRIGHTEN trial described above, 796 patients who did not receive iron supplementation during weeks 0-24 were included, as this study aimed to identify patients who required iron supplementation. Additionally, to exclude the effect of reticulocytosis on the MCV immediately after DA initiation, only the data from weeks 12 to 24 were analyzed.

Diagnostic performance analysis: diagnosing iron deficiency according to MCV and its change

The predictors were MCV at week 24 and changes in MCV from weeks 12 to 24. The MCV represents the average volume of an RBC (fL). It is calculated by dividing the hematocrit (%) by the RBC count (10⁴/μL) and then multiplying the value by 1000. MCV change was included as a predictor based on the hypothesis that a decline in MCV may indicate iron deficiency. A prior study showed a significant increase in MCV after iron supplementation in patients who undergo dialysis [11], supporting its possible utility as a predictor of iron status. Iron deficiency at week 24 was defined as follows: (1) ferritin levels < 100 ng/mL, (2) TSAT < 20%, and (3) either ferritin levels <100 ng/mL or TSAT < 20% [14,15]. TSAT (%) was calculated by dividing Fe by TIBC and multiplying the value by 100.

Predictive performance analysis: predicting iron deficiency after 12 weeks based on ferritin level and transferrin saturation

The secondary analysis was limited to patients without iron deficiency at week 12 to evaluate the performance of ferritin level and TSAT at week 12 in predicting new-onset iron deficiency at week 24. The same three definitions of iron deficiency were used at week 24.

Statistical analyses

Descriptive Analyses

Continuous variables were summarized as means with standard deviations (SDs) or medians with interquartile ranges, whereas categorical variables were expressed as counts and percentages. Baseline characteristics were described at week 12 for all patients and those eligible for the predictive performance analysis.

Diagnostic Performance Analysis

Diagnosing iron deficiency based on MCV and MCV change: The diagnostic performance was assessed using the receiver operating characteristic (ROC) curve and area under the curve (AUC). The AUC ranges from 0.5 to 1, with higher values indicating better performance [16]. The optimal cutoff values were determined using the point at which the Youden index (J index) was maximized [17]. Sensitivity, specificity, positive and negative likelihood ratios, and positive and negative predictive values were calculated using multiple cutoff values. The likelihood ratio indicates the extent to which a given diagnostic test result increases or decreases the pretest probability of the target disorder. A likelihood ratio > 10 or < 0.1 was considered large, and a ratio between 5 and 10 or between 0.1 and 0.2 was considered moderate [18].

Predictive Performance Analysis

Predicting iron deficiency after 12 weeks based on ferritin level and TSAT: Predictive performance was assessed using the ROC curve and AUC. The optimal cutoff values were estimated using Youden’s index [17].

All eligible patients were planned to be included in the analysis, and no prior sample size calculation was conducted. Missing values were not imputed. The analysis was based on estimation, and 95% confidence intervals (CIs) were calculated, but no significance test was conducted. Analyses were performed using SAS, version 9.4 (SAS Institute Inc., Cary, North Carolina), and R, version 4.3.1 (R Foundation for Statistical Computing, Vienna, Austria).

## Results

Participants’ characteristics 

**Figure 1:**
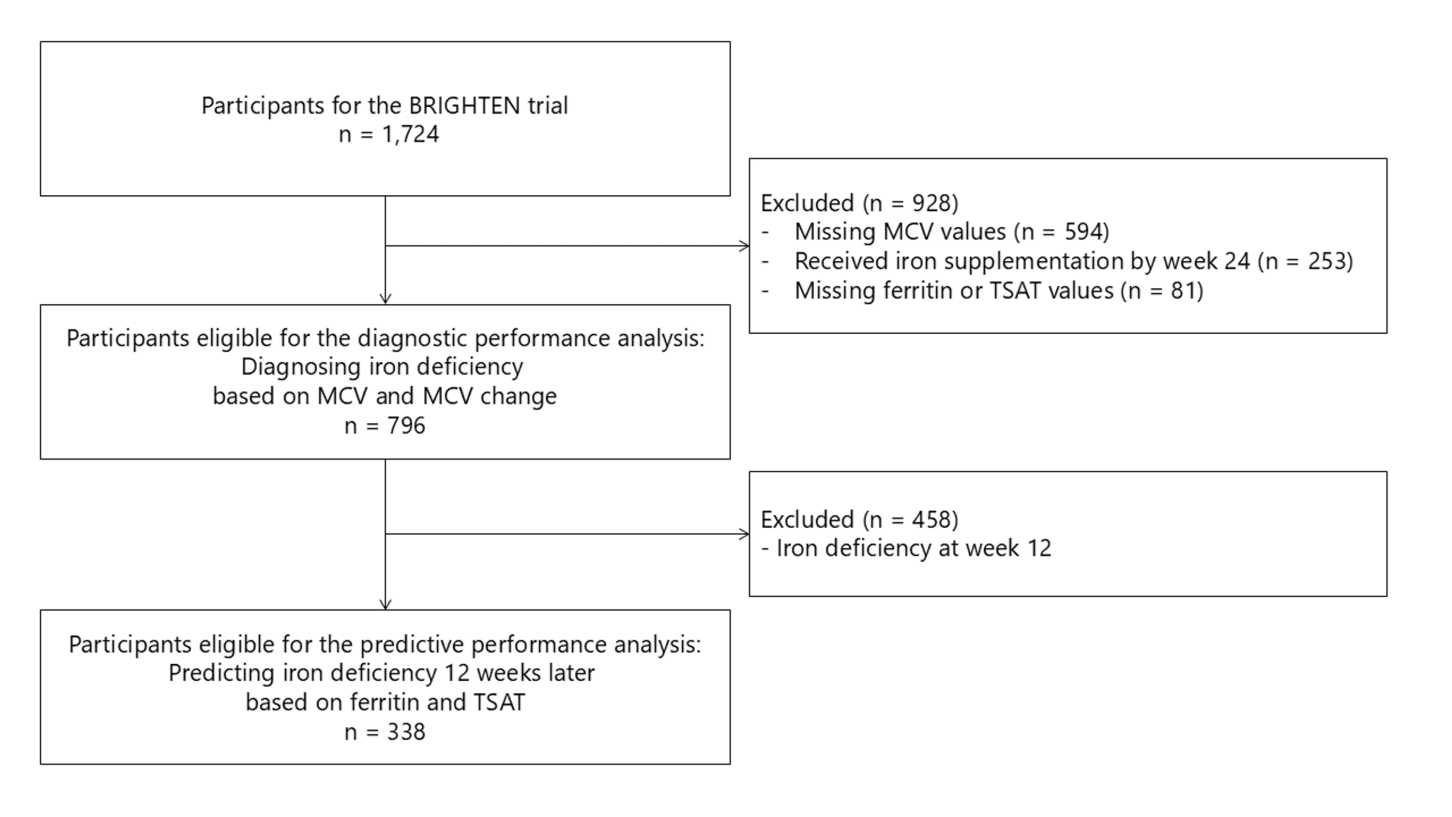
Study flow MCV, mean corpuscular volume; TSAT, transferrin saturation; BRIGHTEN, oBservational clinical Research In chronic kidney disease patients with renal anemia: renal proGnosis in patients with Hyporesponsive anemia To Erythropoiesis-stimulating agents, darbepoetiN alfa.

Of the 1980 patients enrolled in the BRIGHTEN trial across 168 facilities, 256 were excluded, primarily owing to insufficient data on hemoglobin values at weeks 0 and 12. Among the remaining 1,724 patients, 796 were eligible for the substudy of the diagnostic performance analysis of MCV and MCV changes in diagnosing iron deficiency at week 24 (Figure 1). The 338 patients without iron deficiency at week 12 were eligible for the substudy of the predictive performance analysis of ferritin levels and TSAT in predicting iron deficiency after 12 weeks.

The patient characteristics are presented in Table 1. Regarding the diagnostic performance analysis group, the mean age was 70.0 (SD, 11.2) years, 502 participants (63.1%) were men, the mean eGFR was 19.2 (SD, 9.8) mL/min/1.73 m^2^, and mean dose of DA was 50.0 (SD, 30.4) µg/week. Regarding the predictive performance analysis group, the characteristics were similar, except that the ferritin levels and proportions of men and ex-smokers were higher.

**Table 1:** Patient characteristics Data from week 12 are summarized. Continuous variables are presented as means (standard deviations) or medians (interquartile ranges), whereas categorical variables are presented as frequencies (percentages). RAS, renin–angiotensin–system; MCV, mean corpuscular volume; eGFR, estimated glomerular filtration rate; CRP, C-reactive protein; TIBC, total iron-binding capacity; TSAT, transferrin saturation.

Variables	Diagnostic performance analysis (n = 796)	Predictive performance analysis (n = 338)
Age, years	70.0 (11.2)	70.3 (11.5)
Sex, male	502 (63.1%)	251 (74.3%)
Body mass index (kg/m^2^)	23.1 (3.9)	23.5 (3.9)
Cause of kidney disease		
Diabetic nephropathy	222 (27.9%)	100 (29.6%)
Nephrosclerosis	205 (25.8%)	93 (27.5%)
Chronic glomerulonephritis	187 (23.5%)	73 (21.6%)
Others	182 (22.9%)	72 (21.3%)
Smoking status		
Never	366 (46.0%)	123 (36.4%)
Ex-smoker	309 (38.8%)	158 (46.7%)
Current smoker	91 (11.4%)	47 (13.9%)
Unknown	30 (3.8%)	10 (3.0%)
Comorbidities		
Hypertension	764 (96.0%)	328 (97.0%)
Dyslipidemia	447 (56.2%)	193 (57.1%)
Stroke	90 (11.3%)	45 (13.3%)
Ischemic heart disease	128 (16.1%)	53 (15.7%)
Peripheral vascular disease	76 (9.5%)	43 (12.7%)
Malignancy	83 (10.4%)	29 (8.6%)
Collagen or rheumatic diseases	58 (7.3%)	25 (7.4%)
Medication		
Antihypertensives, yes	719 (90.3%)	310 (91.7%)
RAS inhibitor, yes	543 (68.2%)	231 (68.3%)
Antihyperglycemic agents, yes	251 (31.5%)	113 (33.4%)
Immunosuppressive agent or steroid, yes	77 (9.7%)	25 (7.4%)
Laboratory data		
Hemoglobin (g/dL)	11.1 (1.0)	11.0 (1.0)
MCV (fL)	93.0 (5.1)	93.5 (5.3)
eGFR (mL/min/1.73 m^2^)	19.2 (9.8)	19.1 (9.5)
45–59	17 (2.1%)	5 (1.5%)
30–44	82 (10.3%)	34 (10.1%)
15–29	396 (49.7%)	177 (52.4%)
<15	301 (37.8%)	122 (36.1%)
Albumin (g/dL)	3.8 (0.49)	3.7 (0.52)
High sensitivity CRP (ng/mL)	562 (240–1710)	649 (264–2065)
Folic acid (ng/mL)	8.5 (11.1)	8.4 (15.3)
Vitamin B12 (pg/mL)	406 (218)	409 (213)
Fe (μg/dL)	80.8 (25.7)	80.7 (25.0)
Ferritin (ng/mL)	87 (47, 165)	154 (111, 226)
TIBC (μg/dL)	272 (48)	253 (42)
TSAT (%)	30.2 (10.1)	32.1 (9.7)
Protein-to-creatinine ratio (g/gCr)	2.6 (3.1)	2.8 (3.2)
Weekly dose of darbepoetin alpha (μg)	50.0 (30.4)	48.6 (29.5)
None	91 (11.4%)	38 (11.2%)
<30 μg	56 (7.0%)	24 (7.1%)
30–40 μg	265 (33.3%)	120 (35.5%)
40–60 μg	77 (9.7%)	35 (10.4%)
≤60 μg	307 (38.6%)	121 (35.8%)

Changes in hemoglobin, MCV, ferritin, and TSAT over time

**Figure 2:**
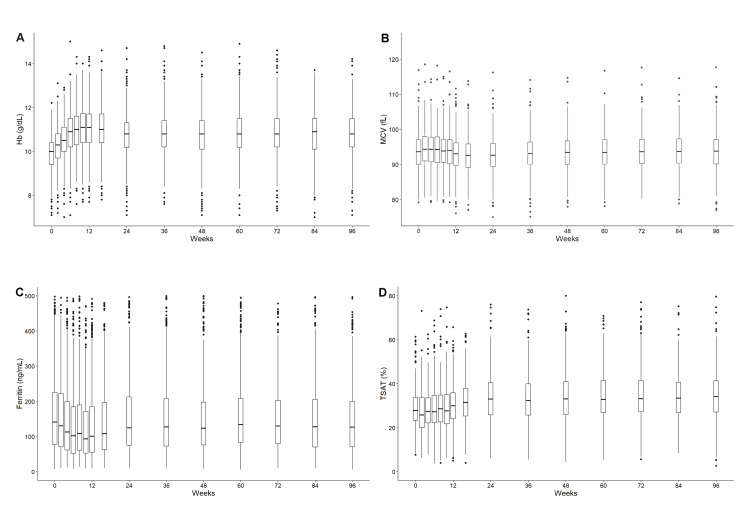
Changes in hemoglobin, MCV, ferritin, and TSAT over time (A) Hemoglobin, (B) MCV, (C) ferritin, (D) TSAT. Hb, hemoglobin; MCV, mean corpuscular volume; TSAT, transferrin saturation.

During the course of the BRIGHTEN trial, a cohort study of ESA administration to patients with ND-CKD, we checked the anemia-related parameters. Hemoglobin levels increased until week 12 (median, 10.0 g/dL at week 0 and 11.1 g/dL at week 12), remaining stable thereafter at a median of approximately 11 g/dL (Figure 2A). The MCV slightly increased until week 6 (median, 93.6 fL at week 0 and 94.4 fL at week 6), then slightly decreased and remained stable at a median of approximately 93.0 fL thereafter (Figure 2B). Ferritin levels decreased until week 10 (median, 147.2 ng/mL at week 0 and 95.0 ng/mL at week 10), subsequently increasing around week 24 and remaining stable at a median of approximately 130 ng/mL (Figure 2C). TSAT remained stable at a median of approximately 27% from weeks 0 to 10, increasing at around week 24 and remaining stable at a median of approximately 33% (Figure 2D). Notably, this population may have begun receiving iron supplementation after week 24.

MCV and MCV changes in diagnosing iron deficiency at week 24

**Figure 3:**
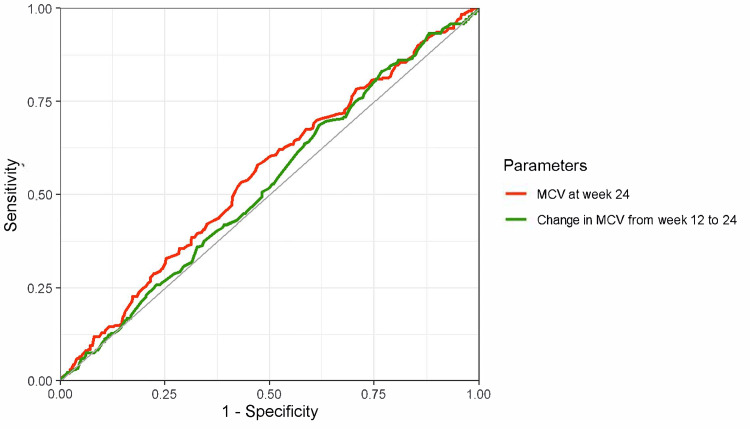
ROC curves of MCV and MCV change for diagnosing iron deficiency (ferritin < 100 ng/mL) Red line: MCV at week 24, AUC 0.55 (95% CI, 0.51–0.59), a cutoff value based on the Youden index 92.9 fL (sensitivity, 58%; specificity, 53%). Green line: MCV change from weeks 12 to 24, AUC 0.52 (95% CI, 0.48–0.57), a cutoff value based on the Youden index 0.40 (sensitivity, 69%; specificity, 38%). The outcome was iron deficiency at week 24, defined as a ferritin level of < 100 ng/mL. ROC, receiver operating characteristic; MCV, mean corpuscular volume; AUC, area under the curve; CI, confidence interval.

**Figure 4:**
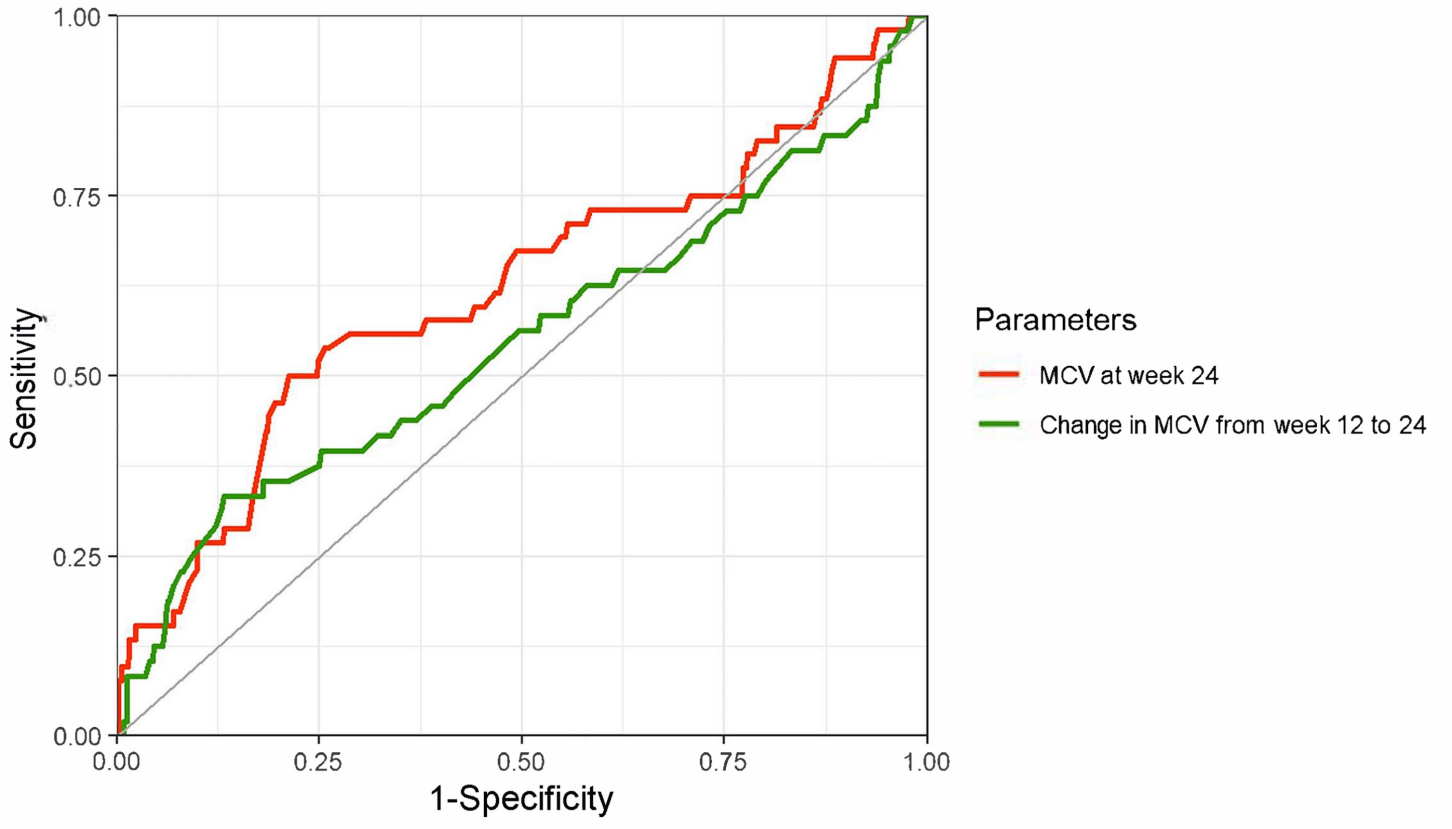
ROC curves of MCV and MCV change for diagnosing iron deficiency (TSAT < 20%) Red line: MCV at week 24; AUC of 0.62 (95% CI, 0.53–0.71), a cutoff value based on the Youden index 88.8 fL (sensitivity, 50%; specificity, 79%). Green line: MCV changes from weeks 12 to 24; AUC of 0.55 (95% CI, 0.45–0.65), a cutoff value based on the Youden index 1.85 fL (sensitivity, 33%; specificity, 87%). The outcome was iron deficiency at week 24, defined as TSAT < 20%. ROC, receiver operating characteristic; MCV, mean corpuscular volume; AUC, area under the curve; CI, confidence interval.

**Figure 5:**
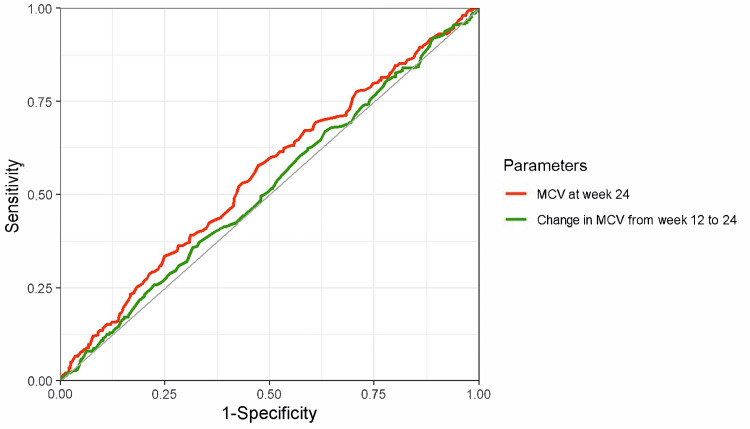
ROC curves of MCV and MCV change for diagnosing iron deficiency (ferritin < 100 ng/mL or TSAT < 20%) Red line: MCV at week 24; AUC of 0.55 (95% CI, 0.51–0.60), a cutoff value based on the Youden index 92.9 fL (sensitivity, 58%; specificity, 53%). Green line: MCV change from weeks 12 to 24; AUC of 0.52 (95% CI, 0.47–0.56), a cutoff value based on the Youden index –1.2 fL (sensitivity, 36%; specificity, 68%). The outcome was iron deficiency at week 24, defined as ferritin < 100 ng/mL or TSAT < 20%. ROC, receiver operating characteristic; MCV, mean corpuscular volume; TSAT, transferrin saturation; AUC, area under the curve; CI, confidence interval.

The ROC curves and diagnostic performance metrics for MCV at week 24 and the changes in MCV from week 12 to 24 for diagnosing iron deficiency (defined as ferritin < 100 ng/mL) at week 24 are shown in Figure 3. The AUCs were low: 0.55 (95% CI, 0.51-0.59) for MCV at week 24 and 0.52 (95% CI, 0.48-0.57) for the change in MCV from weeks 12 to 24. Other diagnostic performance indicators at various cutoff values are summarized in Table 2. The likelihood ratio also suggests limited diagnostic performance of these parameters. When iron deficiency was defined using TSAT < 20% (Figure 4) and TSAT < 20% or ferritin < 100 ng/mL (Figure 5), relatively better performance was observed for TSAT < 20%, although the overall diagnostic performance remained low.

**Table 2:** Diagnostic performance of MCV and its change at various cutoff values for diagnosing iron deficiency (ferritin < 100 ng/mL) MCV, mean corpuscular volume.

	Below the cutoff values		Above the cutoff values							
Cutoff value	Iron deficiency				Sensitivity (%)	Specificity (%)	Likelihood ratio		Predictive value	
	(+) n	(–) n	(+) n	(–) n			Positive	Negative	Positive (%)	Negative (%)
MCV (week 24)										
110 fL	295	473	0	1	100.0	0.2	1.00	0.00	38.4	100.0
105 fL	295	467	0	7	100.0	1.5	1.01	0.00	38.7	100.0
100 fL	276	426	19	48	93.6	10.1	1.04	0.64	39.3	71.6
95 fL	212	322	83	152	71.9	32.1	1.06	0.88	39.7	64.7
90 fL	100	132	195	342	33.9	72.2	1.22	0.92	43.1	63.7
85 fL	24	29	271	445	8.1	93.9	1.33	0.98	45.3	62.2
80 fL	4	4	291	470	1.4	99.2	1.61	0.99	50.0	61.8
75 fL	2	1	293	473	0.7	99.8	3.21	1.00	66.7	61.7
70 fL	0	1	295	473	0.0	99.8	0.00	1.00	0.0	61.6
MCV change (weeks 12–24)										
5 fL	263	430	4	4	98.5	0.9	0.99	1.63	38.0	50.0
4 fL	259	420	8	14	97.0	3.2	1.00	0.93	38.1	63.6
3 fL	256	405	11	29	95.9	6.7	1.03	0.62	38.7	72.5
2 fL	244	378	23	56	91.4	12.9	1.05	0.67	39.2	70.9
1 fL	217	329	50	105	81.3	24.2	1.07	0.77	39.7	67.7
0 fL	164	246	103	188	61.4	43.3	1.08	0.89	40.0	64.6
–1 fL	108	163	159	271	40.4	62.4	1.08	0.95	39.9	63.0
–2 fL	57	84	210	350	21.3	80.6	1.10	0.98	40.4	62.5
–3 fL	28	44	239	390	10.5	89.9	1.03	1.00	38.9	62.0
–4 fL	13	20	254	414	4.9	95.4	1.06	1.00	39.4	62.0
–5 fL	8	14	259	420	3.0	96.8	0.93	1.00	36.4	61.9
–6 fL	4	5	263	429	1.5	98.8	1.30	1.00	44.4	62.0
–7 fL	1	2	266	432	0.4	99.5	0.81	1.00	33.3	61.9

Ferritin levels and TSAT in predicting iron deficiency after 12 weeks

**Figure 6:**
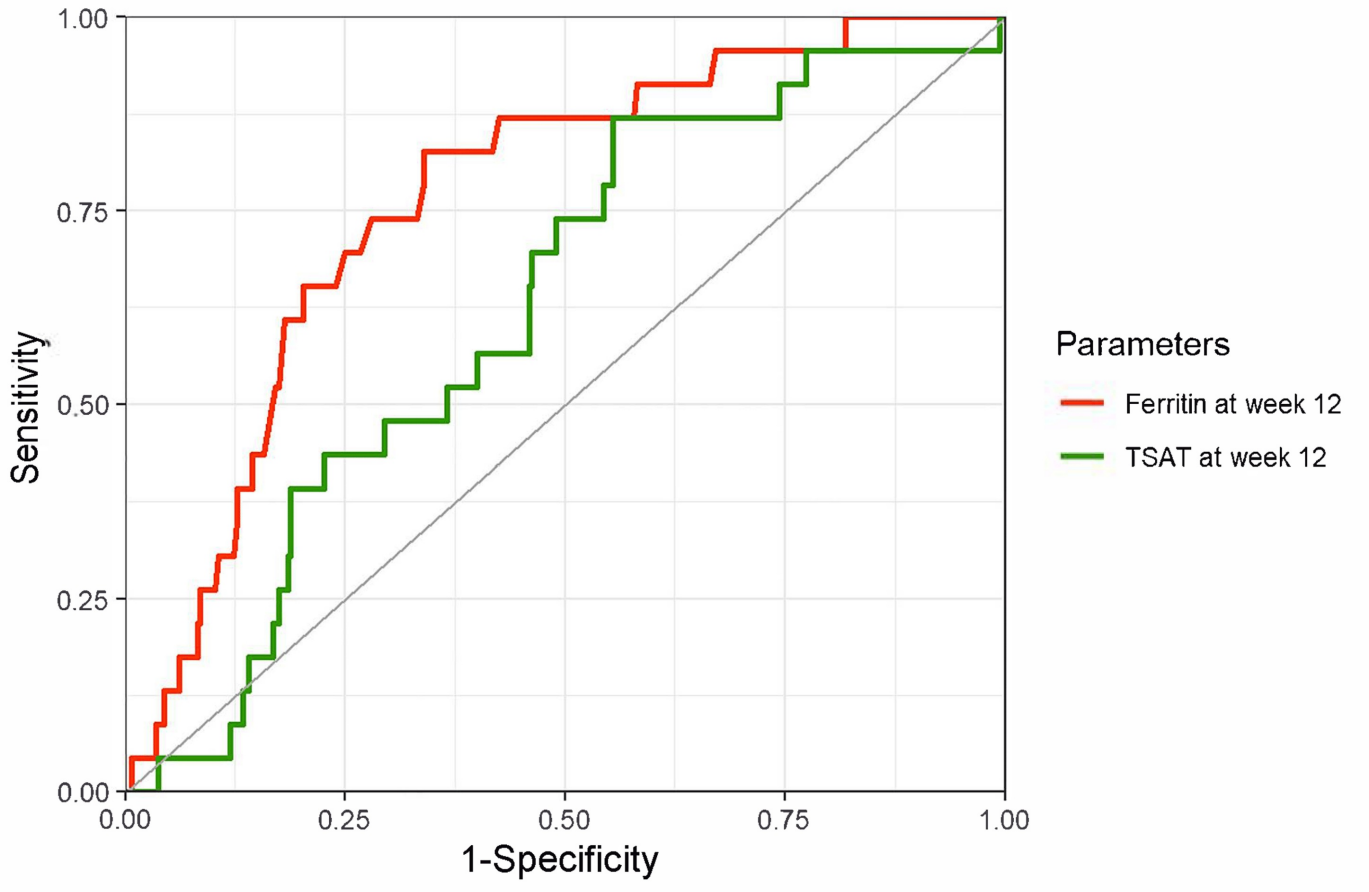
ROC curves of ferritin and TSAT for predicting iron deficiency (ferritin < 100 ng/mL) after 12 weeks Red line: Ferritin at week 12, AUC 0.77 (95% CI, 0.67–0.86), a cutoff value based on the Youden index 131.5 ng/mL (sensitivity, 83%; specificity, 66%). Green line: TSAT at week 12, AUC 0.62 (95% CI, 0.52–0.73), a cutoff value based on the Youden index 33.0% (sensitivity, 87%; specificity, 45%). The outcome was iron deficiency at week 24, defined as a ferritin level of < 100 ng/mL. ROC, receiver operating characteristic; TSAT, transferrin saturation; AUC, area under the curve; CI, confidence interval.

**Figure 7:**
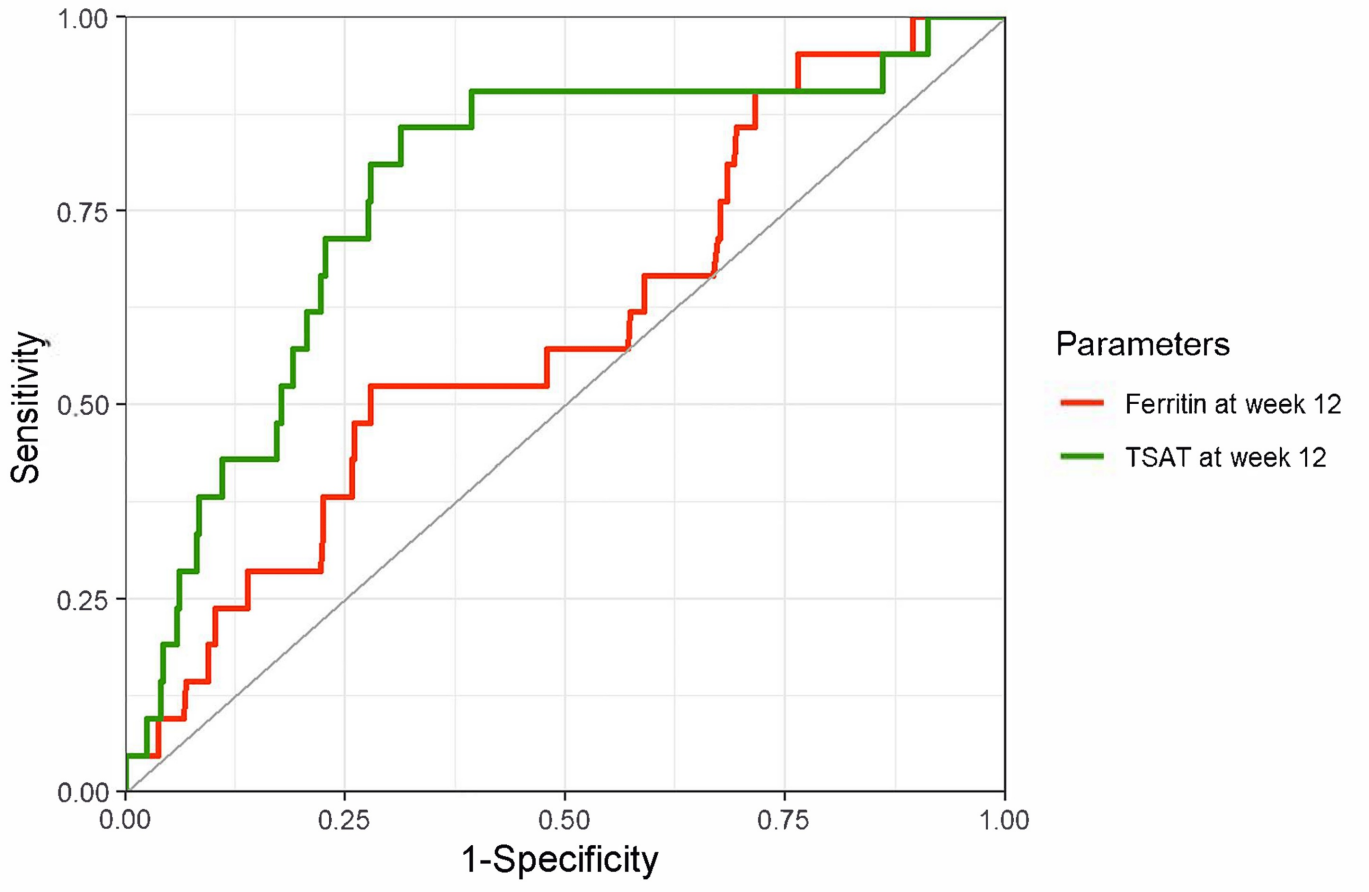
ROC curves of ferritin and TSAT for predicting iron deficiency (TSAT < 20%) after 12 weeks Red line: Ferritin at week 12; AUC of 0.60 (95% CI, 0.47–0.72), a cutoff value based on the Youden index 59.1 ng/mL (sensitivity, 52%; specificity, 72%). Green line: TSAT at week 12; AUC of 0.78 (95% CI, 0.67–0.88), a cutoff value based on the Youden index 28.8% (sensitivity, 86%; specificity, 69%). The outcome was iron deficiency at week 24, defined as TSAT < 20%. ROC, receiver operating characteristic; TSAT, transferrin saturation; AUC, area under the curve; CI, confidence interval.

**Figure 8:**
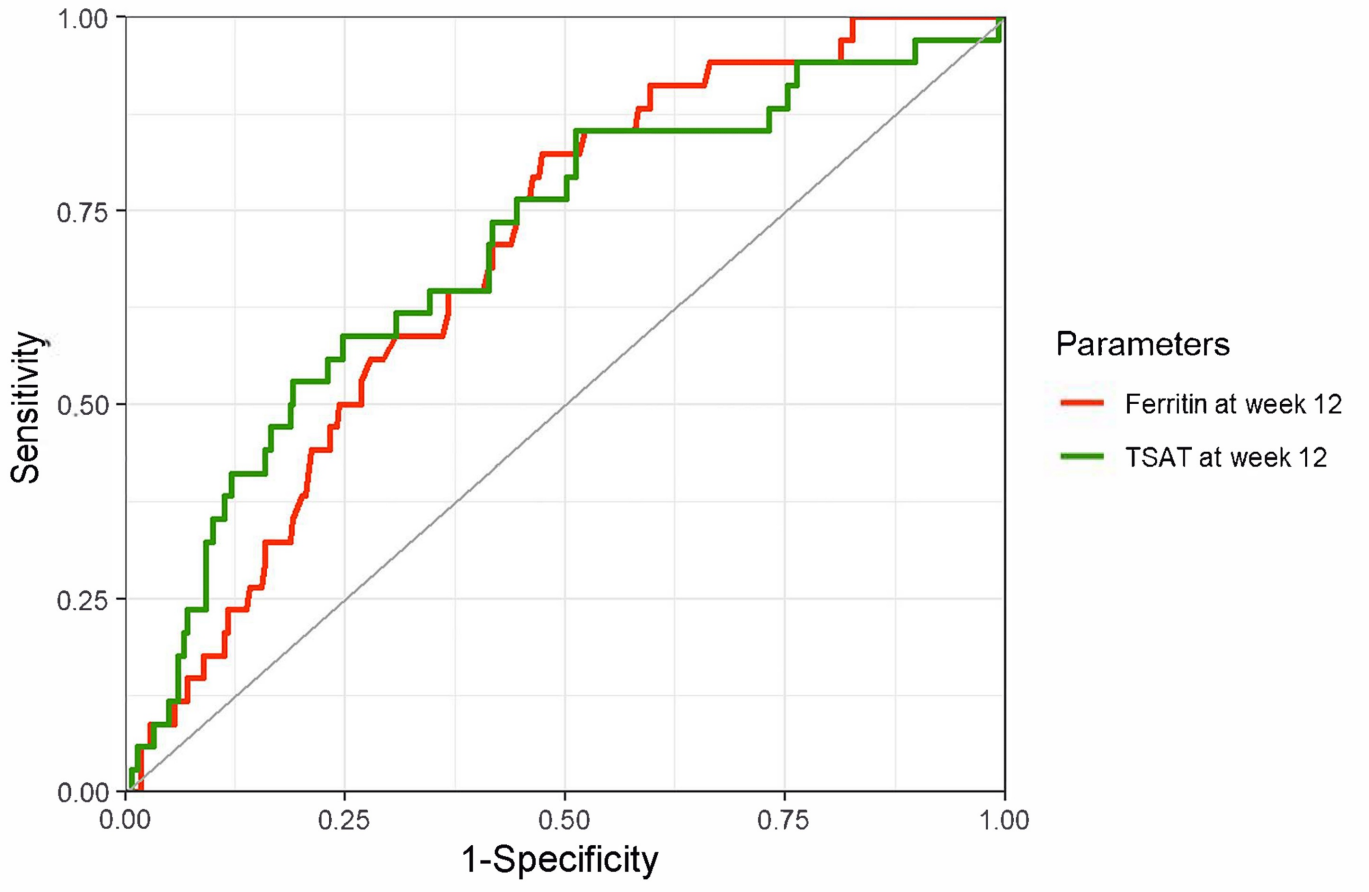
ROC curves of ferritin and TSAT for predicting iron deficiency (ferritin < 100 ng/mL or TSAT < 20%) 12 weeks later Red line: Ferritin at week 12; AUC of 0.69 (95% CI, 0.61–0.77), a cutoff value based on the Youden index 154.5 ng/mL (sensitivity, 82%; specificity, 53%). Green line: TSAT at week 12; AUC of 0.70 (95% CI, 0.61–0.80), a cutoff value based on the Youden index 27.9% (sensitivity, 59%; specificity, 75%). The outcome was iron deficiency at week 24, defined as ferritin < 100 ng/mL or TSAT < 20%. ROC, receiver operating characteristic; TSAT, transferrin saturation; AUC, area under the curve; CI, confidence interval.

The ROC curves and predictive performance metrics for ferritin levels and TSAT at week 12 for predicting iron deficiency (defined as ferritin < 100 ng/mL) at week 24 are shown in Figure 6. The AUCs were higher for ferritin at week 12: 0.77 (95% CI, 0.67-0.86) for ferritin and 0.62 (95% CI, 0.52-0.73) for TSAT. When iron deficiency was defined using TSAT < 20% (Figure 7) and TSAT < 20% or ferritin < 100 ng/mL (Figure 8), better performance was observed when the predictors and index for outcome definition were the same.

## Discussion

To our knowledge, this study is the first to investigate the diagnostic performance of MCV for iron deficiency in patients with ND-CKD and anemia. Among the 796 included patients, MCV and changes in MCV from 12 weeks prior showed minimal diagnostic performance, highlighting the importance of measuring iron status, including ferritin levels and TSAT. We also explored the performance of ferritin and TSAT in predicting iron deficiency after 12 weeks, with ferritin ≤130 ng/mL and TSAT of 33% as suggested cutoff values.

The changes in hemoglobin, MCV, ferritin, and TSAT levels from the start of DA administration are shown in Figure 2. The MCV values initially increased slightly, possibly reflecting reticulocytosis, as reticulocytes have greater volume (120-140 fL) than mature RBCs [19]. All parameters stabilized after week 12. This study focused on patients with ND-CKD who were relatively stable on maintenance ESA therapy; hence, the period from weeks 12 to 24 was selected for analysis. We found that neither MCV nor MCV change was a reliable predictor of iron deficiency. These findings are consistent with those of previous studies involving patients undergoing dialysis [12,20,21], possibly owing to factors that cause an increase in the MCV [8-10], which are more prevalent in patients with CKD than in other populations. A previous study found that among iron-deficient patients, those with an MCV < 85 fL showed a larger reduction in ESA dose after iron supplementation [11]. MCV can serve as a predictor of the response to iron if the population is limited to patients with confirmed iron deficiency based on ferritin or TSAT.

The prevalence of anemia is high in patients with CKD. A previous report found that 37.1% of the patients with stage G4 disease and 52.9% with stage G5 disease had anemia, defined as hemoglobin ≤ 11.0 g/dL or the use of ESAs [22]. Anemia in CKD is associated with a reduced quality of life, need for kidney replacement therapy, and increased mortality [23-25], highlighting the importance of proper anemia management and iron deficiency assessment. The Japanese clinical practice guidelines recommend the evaluation of iron status in patients with CKD and anemia, although the frequency is not explicitly specified [15]. In contrast, the Kidney Disease: Improving Global Outcomes guidelines recommend evaluating the iron status at least every 3 months during ESA therapy [7]. The Japanese guidelines for hemodialysis also suggest evaluation once every 3 months for patients not receiving iron administration [6]. The results of this study underscore the importance of alerting physicians to evaluate and exclude iron deficiency by directly checking iron status (ferritin and TSAT) in patients with ND-CKD.

The strength of this study is that the sample size was relatively larger than that of previous studies, which enabled us to address the research question in the form of a diagnostic performance study. This study has some limitations. First, we included patients without iron supplementation until week 24 to exclude the effects of iron supplementation. This criterion could have led to selection bias; for example, patients for whom physicians may have avoided iron supplementation, such as those with inflammation, may have been more likely to be included. Second, we defined iron deficiency based on ferritin level and TSAT, which have limitations, such as being affected by inflammation and diurnal variation, respectively [26]. Previous studies have defined iron deficiency as an increase in hemoglobin level or a decrease in the ESA dose following iron supplementation [11]. Third, factors that may influence MCV, such as age [8], reticulocytosis [9], malnutrition [10], and other comorbidities such as hepatic disease, were not considered in this analysis. Stratifying or excluding patients based on parameters related to these factors may improve the diagnostic performance of MCV but would reduce the sample size and limit both the feasibility of analysis and generalizability to broader populations. Fourth, this study is a secondary analysis of data collected in a previous study. Although the available variables were appropriate for the study’s objective, the analysis was limited to patients receiving ESA. Additionally, other potential predictors measured in CBC, such as mean corpuscular hemoglobin (MCH) [27], MCH concentration (MCHC) [20], and RBC distribution width (RDW) [21], could not be evaluated because of data collection limitations. Fifth, external validation is required to confirm the diagnostic or prognostic performance of the predictors and the optimal cutoff values.

## Conclusions

MCV and changes in MCV are not reliable predictors in diagnosing iron deficiency among patients with ND-CKD and anemia. This underscores the importance of assessing iron status, such as ferritin levels or TSAT, to accurately identify iron deficiency and optimize anemia management. Ferritin ≤ 130 ng/mL and TSAT ≤ 33% may serve as the optimal cutoff values for predicting new-onset iron deficiency after 12 weeks. These cutoff values could facilitate early intervention before iron deficiency progresses. Further studies are required to assess the performance of other potential predictors, such as MCH, MCHC, and RDW, and alternative definitions of iron deficiency.
